# Alterations of renal phenotype and gene expression profiles due to protein overload in NOD-related mouse strains

**DOI:** 10.1186/1471-2369-6-17

**Published:** 2005-12-21

**Authors:** Karen HS Wilson, Richard A McIndoe, Sarah Eckenrode, Laurence Morel, Anupam Agarwal, Byron P Croker, Jin-Xiong She

**Affiliations:** 1 Center for Biotechnology and Genomic Medicine, Medical College of Georgia, 1120 15^th ^Street, PV6B108, Augusta, GA 30912-2400, USA; 2 Department of Pathology, Immunology and Laboratory Medicine, University of Florida, Gainesville, FL 32610, USA; 3 North Florida/South Georgia Veterans Health System, Gainesville, FL 32608, USA; 4 MD Division of Nephrology, ZRB 614, University of Alabama at Birmingham, 1530 3rd Avenue South Birmingham, AL 35294, USA; 5 The Royal Swedish Academy of Sciences, Kristinebergs Marina Forksningsstation, Fiskebackskil, SE-45034, Sweden

## Abstract

**Background:**

Despite multiple causes, Chronic Kidney Disease is commonly associated with proteinuria. A previous study on Non Obese Diabetic mice (NOD), which spontaneously develop type 1 diabetes, described histological and gene expression changes incurred by diabetes in the kidney. Because proteinuria is coincident to diabetes, the effects of proteinuria are difficult to distinguish from those of other factors such as hyperglycemia. Proteinuria can nevertheless be induced in mice by peritoneal injection of Bovine Serum Albumin (BSA). To gain more information on the specific effects of proteinuria, this study addresses renal changes in diabetes resistant NOD-related mouse strains (NON and NOD.B10) that were made to develop proteinuria by BSA overload.

**Methods:**

Proteinuria was induced by protein overload on NON and NOD.B10 mouse strains and histology and microarray technology were used to follow the kidney response. The effects of proteinuria were assessed and subsequently compared to changes that were observed in a prior study on NOD diabetic nephropathy.

**Results:**

Overload treatment significantly modified the renal phenotype and out of 5760 clones screened, 21 and 7 kidney transcripts were respectively altered in the NON and NOD.B10. Upregulated transcripts encoded signal transduction genes, as well as markers for inflammation (Calmodulin kinase beta). Down-regulated transcripts included FKBP52 which was also down-regulated in diabetic NOD kidney. Comparison of transcripts altered by proteinuria to those altered by diabetes identified mannosidase 2 alpha 1 as being more specifically induced by proteinuria.

**Conclusion:**

By simulating a component of diabetes, and looking at the global response on mice resistant to the disease, by virtue of a small genetic difference, we were able to identify key factors in disease progression. This suggests the power of this approach in unraveling multifactorial disease processes.

## Background

The cause of the relentless progression of chronic kidney disease (CKD) to chronic renal failure is likely to be multifactorial. CKD itself has a variety of inciting etiologies [1]. Accumulating clinical evidence indicates that proteinuria is associated with CKD [2] and predictive of progression in CKD regardless of diverse etiologies [3]. Subsequent animal studies and *in vitro *experiments provided additional evidence for proteinuria in progressive CKD. Because kidney complications occur frequently in diabetic patients, we recently conducted a microarray study on gene transcripts altered by diabetes in non-obese diabetic (NOD) mice kidneys [4]. As NOD spontaneously develops both diabetes and proteinuria, our previous studies could not distinguish gene expression changes due to diabetes versus proteinuria. To identify gene expression changes due to proteinuria alone, we investigate gene expression changes in two NOD-related mouse strains (NON and NOD.B10) that are diabetes resistant but can nevertheless develop proteinuria if subjected to protein overload by peritoneal injection of Bovine Serum Albumin (BSA). The protein overload model is a conceptually simple, clear and direct model [5,6] to demonstrate the pathogenicity of proteinuria. The use of NON and NOD.B10 serves as a control for the differences due to genetic background, as NOD.B10 differs from NOD by the MHC genes and NON differs from NOD for approximately 30–40% of the polymorphic markers. Our studies demonstrate a number of gene expression changes induced by protein overload. Some of them appear to be related to the signaling pathways in proteinuria. These studies confirm suspected therapeutic targets for prevention of CKD.

## Methods

### Animals

NON and NOD.B10 mice were obtained from Jackson Laboratories, Bar Harbor, ME. All animal studies were approved by the University of Florida's IUCAC prior to the start of these experiments. Two mouse strains were assayed for gene expression profile changes due to protein overload. Data was collected from 5 – 7 animals per strain. Male 8 – 10 weeks old NOD.B10 and NON mice were given intraperitoneal (IP) injections of bovine serum albumin (BSA, 1 g/100 g body weight) (CAT# A-7906; Sigma Chemical Company, St. Louis, MO, USA) in saline (25% solution weight/volume) or normal saline 5 days a week, for 10 weeks and sacrificed 10 weeks post treatment. We extended the injection protocol by four weeks compared to previous studies [6,7]. The reference RNA for micorarray hybridization was made from a pool of total RNA obtained from eight 10 week non-diabetic female NOD kidneys and described previously [4].

### Tissue preservation and histology

Immediately after sacrificing mice, the kidneys were collected, flash frozen in liquid nitrogen and stored at -80°C until RNA extraction. At terminal surgery for sacrifice, the two poles of the right kidney were removed, snap frozen, and held at -80°C for RNA extraction. Kidney sections were stained using periodic acid Schiff (PAS). Histologic features of mesangial expansion or Intercapillary glomerulosclerosis as defined by Kimmelstiel and Wilson (1936) [8] were graded for comparison using the following glomerulosclerosis score (GS): none = 0, stalk glomerulopathy = 1 [9], segmental intercapillary = 2, diffuse intercapillary = 3, and nodular intercapillary = 4. Histologic sections were blind ranked and analyzed by the Wilcoxon rank test for statistical significance.

### Microarray fabrication and hybridization

Total RNA was extracted from kidneys using a midi-kit from Qiagen Co. (Hilden, Germany). The procedure for printing the microarrays, hybridization to the microarrays and analysis was previously described [4]. Briefly, a 5760 clone subtractive cDNA library was prepared and printed on glass slides using the BioRobotics MicroGrid TAS II. The RNA material used to interrogate the microarrays was prepared separately. Total RNA were individually extracted from whole kidneys of mice belonging to each study group and the reference group (described in the previous section). The reference RNA pool was made by combining equal quantities of total RNA from the kidneys of the reference group of mice. Each of the study and reference RNAs were subsequently converted to cDNA and respectively labeled with Cy-3 and Cy-5 fluorophores. Cy-3 labeled probes from each study mouse were then combined to an equal quantity of the universal Cy-5 labeled reference and hybridized to the slides. After scanning the slides at two wavelengths, corresponding to the Cy-3 and Cy-5 dye fluorescence, two images were computer generated and artificially superimposed. Numerical values (expression levels) for each DNA spot on the array were extracted from the images using AnalyzerDG (MolecularWare Cambridge, MA). Each hybridization reaction was repeated twice to ensure reproducibility and confidence in the measurement and the mean values of hybridization signals were used. The data (along with background and error measurements) were stored into a file in the form of expression ratios of the study-sample/reference intensities. Statistical analyses were performed on the expression data in order to find differentially expressed genes that could distinguish treated from untreated animals. Genes showing significantly different expression differences between the compared groups were clustered with Cluster and their expression pattern graphically illustrated using TreeView (as described in [4]).

## Results

### Histopathology

**Figure 1:**
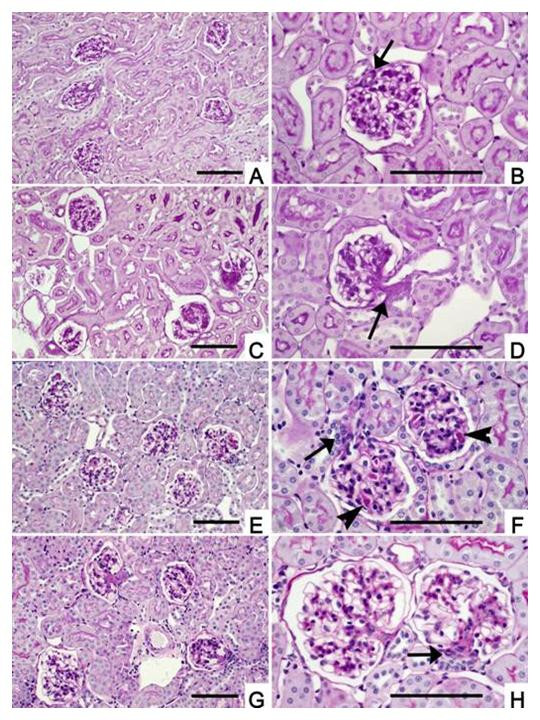
**Histopathology of protein overload kidney from control and experimental animals**. Parts A, C, E, G are all the same magnification (scale bar 0.1 mm) for comparison of relative sizes among groups. Similarly, parts B, D, F, H are all the same magnification (scale bar 0.1 mm) and each figure has the justiglomerular apparatus (JGA) denoted by an arrow head to show the vascular pole. **A) **Survey view showing normal cortex (200×) for NOD.B10 treated with saline (PAS stain). **B) **Higher magnification of saline-treated NOD.B10 showing normal glomerular structure (400×). JGA is shown (arrow) and tubular pole is opposite. **C) **BAS-treated NOD.B10 (PAS stain). Survey view showing increased staining in all 4 glomeruli accentuating Glomerular lobules (200×). **D)**. Higher magnification of a BSA-treated NOD.B10 glomerulus showing expansion extending from the JGA region (arrows) to the peripheral mesangium accentuating the glomerular lobularity (400×). **E) **Saline treated NON (PAS stain) survey view of 5 glomeruli with preserved parenchyma (200×). Glomerular hyaline is just visible. **F)**. Higher magnification of saline-treated NON shows intracapillary hyaline thrombi (arrows, 400×). **G) **BAS-treated NON (PAS stain) survey view (200×) of cortex with 4 glomeruli showing changes similar to those seen in BSA-treated NOD.B10 mice. No residual intracapillary thrombi are present. **H) **Higher power view of two BSA-treated NON glomeruli with varying degrees of mesangial expansion. JGA is indicated by arrow (400×).

Histology features were assessed in control and BSA groups of each strain and summarized by the mean Intercapillary glomerulosclerosis score (GS score). NOD.B10 mice treated with saline have normal histology (GS = 0), while BSA treated mice average GS scores of 3.0 (p < 0.005). Glomeruli from BSA treated mice also show distinct hypertrophy in comparison to controls. This is best appreciated at low magnification (Figure 1a vs. 1c) when a representative population of each sample can be observed. The NON strain presents more complex features (Figure 1e and 1f). The control animals have early features of lipoprotein glomerulopathy as reported previously [10]. The intracapillary hyaline thrombi are visible in most glomeruli (Figure 1f, arrows). Mild mesangeal expansion (GS = 0.6) is also present. Treated NON mice show glomerular hypertrophy (Figure 1g vs. Figure 1e) and increased mesangeal expansion (GS = 3.3, p < 0.005) which is similar in the BSA-treated NOD.B10 group. Coincidentally the intracapillary hyaline deposits are absent from treated NON mice.

### Impact of protein overload on gene expression

#### NON mice

**Figure 2:**
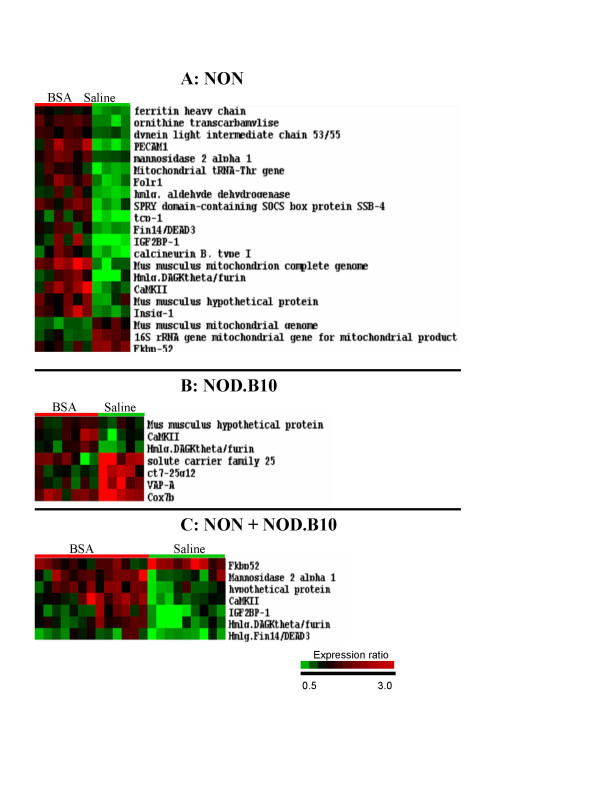
**Microarray profiles of mouse kidneys treated with BSA or saline**. **A) **Microarray profiles in kidneys from NON mice treated with BSA versus saline. Each column in the picture represents the median value of two array experiments for an individual mouse. Each row represents a different gene on the array. The identity of each gene is displayed on the right hand side of the picture. On each row of the picture, the red, green or black colors refer to the relative expression of the studied gene compared to the reference. Green represents lower expression, red represents higher expression and black represents equal levels of expression. The intensity of red and green color is proportional to the increase or decrease of gene expression relative to the reference. **B) **Expression profiles in kidneys from NOD.B10 mice treated with BSA versus saline. **C) **Combined analysis of NON and NOD.B10 treated with BSA versus saline.

Among the 5760 clones screened by microarray, 21 unique transcripts differ between the BSA and saline treated NON mice (Figure 2A and Table 1). Eighteen of these genes are up-regulated and 3 are down-regulated. The up-regulated clones represent genes pertinent to kidney function, such as ornithine transcarbamylase, an enzyme of the urea cycle, and ferritin heavy chain (fth), an enzyme that stores iron in a soluble non-toxic form. The transcript for mannosidase 2 alpha 1 (Man2a1), an enzyme involved in extracellular N-glycan branching, is also up-regulated. The gene for Man2a1 which maps to chromosome (Chr.) 17 on a probable QTL for lymphocytosis [11] has previously been associated with diabetes and autoimmune diseases such as lupus nephritis. In the BSA treated NON mice, Man2a1 is up-regulated 1.4 fold, in a very similar manner to the up-regulation observed in kidneys of db/db mice with type 2 diabetes and albuminuria (1.4–1.7 or 1.8 reported in the literature [12,13]). Some up-regulated transcripts in the BSA treated NON mice suggest recruitment of immune related pathways and inflammation. These transcripts include calmodulin kinase beta (CaMKII), calcineurin B, a regulator of T cell receptor signalling and platelet endothelial adhesion molecule 1 (PECAM-1), which is a potential prognostic marker for leukocyte infiltration [14]. Moreover, in BSA treated NON mice the folate receptor 1 (Folr1) gene and the insulin-induced gene 1 (Insig-1) transcripts increase by 1.8 and 2.1 fold respectively, compared to controls. This is interesting because in kidney mesangial cell cultures, subjected to inflammatory stimuli, Folr1 expression has been shown to increase 2.0 fold, 6 hours after stimulation, while Insig-1 expression increases by 1.9 fold within 2 hours of stimulation [15]. The increase in Insig-1 may additionally point to a role for lipid metabolism during proteinuria. Some BSA up-regulated genes in the NON mice map to noteworthy chromosomal locations. For example, PECAM 1 and the gene for Insulin Growth Factor 2 Binding Protein 1 (Igf2Bp1, also called CRDBP) which protects c-Myc mRNA, from degradation [16] map to mouse Chr. 11. PECAM-1 is located near protein kinase C alpha, an enzyme implicated in albuminuria during type 1 diabetic nephropathy [17]. Moreover, several other genes between PECAM-1 and Igf2Bp1, such as Growth Hormone, STAT3, and STAT5 [18,19] are involved in diabetic nephropathy or diabetes and an identified IDD4 interval spans STAT3 [20]. Down-regulated gene products in BSA treated NON mice encode primarily mitochondrial genes, as well as the immunophilin FKBP-52.

#### NOD.B10 mice

7 transcripts are affected by BSA treatment in NOD.B10 mice (Figure 2B and Table 1). Three are up-regulated, two of which overlap with the up-regulated transcripts in the NON mice (CaMKII and a DAGKθ homologous clone). The third up-regulated transcript is for ribosomal protein S-23 (RpS23), which has also been demonstrated to be up-regulated in mouse kidney following angiotensin II administration [21]. Four transcripts are down-regulated (Figure 2B). Two are mitochondrial genes, (solute carrier family 25 member a3 (Slc25a3) and cytochrome oxidase subunit 7b (Cox7b)) while the two others (acetyl CoA transporter and vesicle-associated membrane protein-associated protein-A (VAPA)), are involved in lipid transport and processing. VAPA, whose gene maps closely on Chr. 17 (63.3 cM) to Man2a1 (62.3 cM) (discussed above), is a syntaxin-like protein implicated in Endoplasmic Reticulum/Golgi vesicle transport and phospholipid regulation in mammalian cells. VAPA was recently found in a complex with Oxycosterol-binding protein (OSBP), a protein involved in sterol homeostasis. The VAPA-OSBP complex intervenes at a stage of protein and lipid export from the ER [22]. Acetyl CoA transporter transfers gangliosides from the ER to the Golgi apparatus for O-acetylation.

### Effect of overload treatment and effect of proteinuria in diabetic nephropathy

To identify genes commonly affected by protein overload, NON and NOD.B10 mouse strains were analyzed jointly (Figure 2C). The effects of proteinuria in diabetic nephropathy are difficult to distinguish from those of other factors such as hyperglycemia. For this reason, the overload induced gene expression changes in NON and NOD.B10 were compared to changes induced by type 1 diabetes in NOD [4]. Several genes seem to be affected by BSA treatment only (Table 1 and Table 2) but noteworthy are the genes for fth, a regulator of oxidative tolerance in the kidney, and Man2a1. For those genes, independent studies tend to confirm key effects of proteinuria. For example, fth expression is not significantly altered by diabetes in NOD mice but it increases by 1.3 fold in NON and NOD.B10 mice treated with BSA. This suggests that fth gene expression is sensitive to proteinuria, a hypothesis which is reinforced by proteomic work on the OVE26 transgenic mouse, another mouse model for type 1 diabetes [23]. Compared to the diabetic NOD mouse, which displays characteristics of early human diabetic nephropathy, the diabetic OVE26 transgenic mouse, displays characteristics significantly closer to advanced human diabetic nephropathy [24], which include notably a dramatic increase of urinary albumin excretion. In diabetic OVE26 mice with proteinuria, the protein level of fth increases by 1.93 fold compared to control mice [23], suggesting, by comparison to NOD and to our present study, that proteinuria is a key factor. Like fth, Man2a1 gene expression does not significantly change in type 1 diabetic NOD mice. However BSA treated NON and NOD.B10 mice are subject to a 1.5 fold increase in expression of this gene. Remarkably, results from a recent genomic profiling study on the impact of type 1 and 2 diabetes on the kidney [13] show that Man2a1 expression does not change significantly in streptozotocin treated mice, a model for type 1 diabetes, when animals do not present advanced mesangial matrix expansion nor albuminuria. In the same study however, Man2a1 is up-regulated 1.4 times in hyperglycemic/hyperalbuminuric mice compared to control mice. Man2a1 is also up-regulated 1.8 fold, in mice with advanced mesangial expansion compared to controls.

The NON mice used in this work, which were more affected by protein overload, experienced additional BSA up-regulation of the transcript for the t-complex 1 (tcp-1). Remarkably Tcp-1 maps to Chr 17 at 11.6 cM in a recently identified QTL for proteinuria (between D17Mit113 (6 cM) and D17Mit46 (15 cM)) [25] indicating that this gene may also be more specifically regulated by proteinuria in the kidney.

Some genes in our study are affected in the same way by protein overload and diabetes, suggesting that their expression is regulated by either mild proteinuria (or proteinuria onset) or alternatively other factors. Such is the case for the immunophilin FKBP-52 transcript which is down-regulated 0.8 fold in the kidney of both mouse strains treated with BSA and diabetic NOD mice.

## Discussion

Proteinuria is a risk factor for progression of diverse causes of CKD that account for two thirds of patient entries into end stage renal disease programs in the US [1]. The largest single cause of progressive CKD is diabetic nephropathy. An analysis of the protein overload model might help dissect mechanisms of pathogenesis that are common to diabetic nephropathy and other etiologies of CKD linked by proteinuria. Furthermore, the power of the analysis would be strengthened by comparing changes in transcription between diabetes and proteinuria without diabetes. The analysis would be facilitated by using animals with similar genetic backgrounds. Similarities in genotype would reduce variances in phenotypic expression which were incidental to pathogenesis. In a previous study we reported the changes seen in early diabetic NOD mice which is a prototypic mouse model of type I diabetes. For the present study we chose two genetically related strains. NON is non-obese non-diabetic, related to NOD by common parentage and shares some of the diabetogenic loci with NOD but significantly its H-2 locus imparts diabetes resistance even on the NOD background [26]. While free of diabetes NON does exhibit lipoprotein glomerulopathy. Extending this principle, the NOD.B10H-2^b ^congenic strain differs only at the H-2 locus and is also diabetes free [27].

The histologic responses to protein overload had similarities in both non-diabetic strains examined here which were more profound than we previously observed for NOD mice coincident with diabetes [4] or the changes described in the db/db mouse model of type II diabetes [28]. Renal hypertrophy of glomeruli and tubules is a common feature of diabetes [18,29,30] as well as other conditions, including high protein diet and compensatory hypertrophy [31]. Similarly glomerulosclerosis with mesangeal expansion is typical of diabetic nephropathy but may also be seen in other conditions [8]. Regardless, the histologic response of NON was more complex with the unexpected resolution of glomerular capillary lipoprotein thrombi. The mechanism of lipoprotein deposition is unclear but the deposits normally increase with age and are characteristic of the glomeruli [10]. This may be related to the relatively unique flow characteristics of the glomerulus. In glomerular hypertrophy flow is increased [29] and may be accompanied by an increase in the number of glomerular capillaries and an increase in their size [32]. Any of these factors may prevent or reverse the formation of intracapillary thrombi. The more complex histopathologic response seen in NON is also reflected at the gene expression level, whereby, in NOD.B10, only 7 transcripts are altered by overload treatment, as opposed to 21 transcripts in the NON model. Some of the common features of BSA treatment in NON and NOD.B10 mice nevertheless transpire through the transcription profiles, whereby BSA upregulated genes in NOD.B10 essentially overlap with those observed in the NON.

### Immune related response

CaMKII is up-regulated in both NON and NOD.B10 treated groups. The major causes of nephropathy in CKD (diabetes and hypertension) have not traditionally been considered autoimmune or even immune mediated. However, in several rodent models, there is increasing evidence that adaptive cellular immunity and innate immunity play a pathogenetic role [33], as well as models that are generally considered idiopathic proteinuria including protein overload [6]. In this context CaMKII, has been shown to modulate the activity of NFκB, a central mediator of immune, inflammatory and stress responses [34]. NON mice, which are most affected by the overload treatment, display the additional up-regulation of calcineurin B and PECAM-1, suggesting a more pronounced leukocyte infiltration of the kidney as noted above. PECAM-1, whose gene is also up-regulated in type 1 diabetic nephropathy [35] participates in leukocyte transmigratory processes and is regulated by modulators of NFκB [36]. Calcineurin B is the regulatory subunit of calcineurin which plays a pivotal role in antigen stimulated T cell activation [33]. Our findings extend the rapid response microarray studies of Nagasawa et. al. [7] to protein overload, which also show involvement of an immune related response, with the up-regulation of osteopontin, a chemotactic and adhesion molecule for macrophages which promotes macrophage infiltration during interstitial fibrosis and wound healing.

### Involvement of Fkbp-52

FKBP-52, an immunophilin, is down-regulated in the BSA treated NON and NOD.B10 mice. FKBP-52 binds to immunosuppressants such as FK506 and is part of the nontransformed glucocorticoid-receptor (GC-receptor). In the kidney cortical collecting duct, maneuvers inactivating FKBP-52 or slowing it's disassociation from the receptor complex, reduce the response of calcineurin to steroid hormones [37]. FKBP-52 can be co-purified with CaMK and is also phosphorylated *in vitro *by CaMK [38]. This has led to suggest that FKBP-52 plays a role in signaling pathways involving phosphorylation by CaMK. Moreover, CaMK inhibitors, as well as immunosuppressants have strictly parallel effects even in different cell types [39]. Taking into account that the transcription of FKBP-52 varies due to BSA-treatment coincidentally with CaMKII, the FKBP-52/CaMKII axis may have a role in the response to BSA-treatment in mice. FKBP-52 may nevertheless have a more diversified role since it is also down-regulated in type I diabetes [4]. In human, the specific target for FKBP-52 is phytanoyl-CoA alpha-hydroxylase (PAHX), an orthologue of mouse LN1, which is potentially involved in the progression of lupus nephritis [40]. PAHX and SCD-1, another enzyme downregulated in NOD diabetic mice, play a sequential role in peroxisomal lipid processing, suggesting that disruptions in lipid homeostasis occur in diabetes and proteinuria.

### Lipid processing

In NOD.B10 and NON mice, BSA treatment affects several transcripts involved in lipid processing. In NON, Insig-1 is up-regulated. When up-regulated, Insig-1 is known to block up-regulation of Peroxisome Proliferator receptor (PPAR) gamma-2 [41]. This is interesting because PPAR-gamma agonists have also been shown to protect against diabetic nephropathy in type 1 diabetes models [42-44] as well as in type 2 diabetes concurrent with obesity [45]. Such agonists also protect against nondiabetic glomerulosclerosis [46]. PPAR gamma expression is known to be under the control of the Signal transducer and activator of transcription (STAT) 5 [47] and it has recently been reported [19] that albumin treatment of proximal tubular cells increases the activity of STATs. We did not detect changes of STATs at the expression level. This could be explained by the absence of the STAT genes on our microarray or a change of STAT activity. The PPAR gamma and janus kinase-signal transducer and activator of transcription (JAK-STAT) signaling pathways may therefore play a role in the response to BSA treatment. Remarkably, STAT 5 proteins can also associate with the GC-receptor in a highly regulated manner [48]. Since glucocorticoids stimulate the expression of PPAR gamma in adipocytes and FKBP-52 is part of the GC-receptor complex, there may be a crosstalk between the glucorticoid receptor, PPAR gamma and JAK-STAT signaling during BSA treatment.

### Possible effects of strain differences

Although the experimental design tries to dissect the effects of proteinuria from the initial source of CKD, some of the responses observed in this study could point out differences between mouse strains. For example, although the GC-receptor may be involved, it may act slightly differently in each strain. In this regard, it is relevant to point out the increase of the Igf2bp1 protein in BSA treated NON mice. Igf2bp1 is known to stabilize c-Myc mRNA, preventing its degradation. It is known that T lymphocytes of NOD mice are resistant to apoptosis induced by glucocorticoids which normally down-regulate c-myc through the activated GC-GC receptor complex. Martins and Aguas [49] investigated whether expression of Myc protein, in response to dexamethasone stimulation, was the same in NOD mice compared to NON and other mice not prone to diabetes. They found a consistent increase in the levels of Myc protein after GC-treatment of lymphocytes of NOD mice, in contrast with the down-regulation of c-myc in lymphocytes from mice not prone to diabetes, suggesting that the GC-receptor may not regulate c-Myc expression in the same way in the NOD and NON mice. We and others [45] also observed that lipid processing and PPAR gamma play a role in kidney complications, and because PPAR gamma and STAT5b are known to auto-regulate each other in a retrocontrol manner [47], we also discussed a possible cross-talk between PPARs and STATs. However, the auto-regulation mechanism of PPAR gamma by STAT5b may be deficient in NOD mice, in light of recent evidence that NOD mice present a mutation in the DNA binding domain of STAT5b, which reduces STAT5b DNA binding affinity [50].

## Conclusion

In summary, this study highlights some of the differentially regulated pathways that may be important in the progression of CKD. Some of the pathways appear to be common to different inciting etiologies and others may be unique as demonstrated by the NON versus NOD.B10 paradigm. By global histologic criterion these two strains appear to have the same end point but they begin at different places. This paradigm is reflected in the gene expression analysis and suggests the power of this approach in unraveling a complex disease process.

## Competing interests

The author(s) declare that they have no competing interests.

## Authors' contributions

KW drafted the outline of this manuscript and contributed to the discussion of the microrray results. She also constructed the substractive library, PCR amplified and purified the 5760 library clones for the array chips, conducted the microarray hybridizations followed by data analysis and sequencing of the clones of interest. JXS contribution is in experimental design, data interpretation and preparation of manuscript. BC's role in the project was to design, supervise and complete the in vivo work and the pathology. LM participated in data acquisition. RMI provided bioinformatics support, and data analysis. AA assisted in the design and data interpretation of the manuscript. SE printed the arrays. All authors have read and approved the manuscript.

## Pre-publication history

The pre-publication history for this paper can be accessed here:



## Figures and Tables

**Table 1:** Expression differences between BSA and saline treatment

**Gene name/Function**	**ID**	**Expression ratios**			**p values**		
		
		T1/C1	T2/C2	T3/C3	T1/C1	T2/C2	T3/C3
**Kidney function**							
Ornithine transcarbamylase (OTC)	[Genbank:X07094]	1	1.7	1	NS	2.66E-05	NS
Folate receptor 1 (Folr1)	[Genbank:XM_133582]	1	1.8	1	NS	4.12E-04	NS
**Lipid Metabolism**							
Insulin induced gene 1 (Insig1)	[Genbank:NM_153526]	1	1.8	1	NS	8.05E-04	NS
VAMP associated protein A (VAPA)	[Genbank:BC003866]	0.7	1	1	2.47E-03	NS	NS
Acetyl-CoA transporter (AcylCoAtrsp)	[Genbank:BC005616]	0.7	1	1	3.13E-02	NS	NS
**Immune**							
Calmodulin-dependent protein kinase beta (CaMKII)	[Genbank:XM_132366]	1.4	1.9	1.6	3.63E-02	4.86E-03	3.23E-04
Homolog human calcineurin B, type I (Calcineurin BI)	[Genbank:BC027913]	1	1.8	1.4	NS	9.99E-04	1.45E-02
Platelet Endothelial Cell Adhesion Molecule 1 (PECAM1)	[Genbank:BC008519]	1	2.1	1	NS	1.98E-04	NS
SPRY domain-containing SOCS box 4 (SSB-4)	[Genbank:NM_145134]	1	1.8	1	NS	4.54E-04	NS
**Signal Transduction**							
Homolog diacylglycerol kinase, theta (HmlgDAGKθ)	[Genbank:NM_199011]	1.3	1.9	1.6	1.93E-02	4.54E-03	3.08E-04
**Glucocorticoid receptor**							
Immunophilin FKBP-52 (FKBP52)	[Genbank:X17069]	1	0.8	0.8	NS	6.81E-03	3.27E-03
**Apoptosis**							
Insulin-like Growth factor2 binding protein1 (IGF2bp1)	[Genbank:XM_109627]	1	2	1.5	NS	9.40E-04	7.71E-04
Homolog apoptosis-related PNAS2 (Hmlg.apopts)	[Genbank:AK008911]	0.9	1	1	4.88E-02	NS	NS
Histone Acetylase subunit MRG15-1 (HistAcetyl)	[Genbank:AF319620]	0.7	1	0.7	4.36E-02	NS	2.07E-02
DEAD/H box polypeptide 3 (DEAD3)	[Genbank:NM_010028]	1	1.6	1.4	NS	8.25E-04	6.26E-03
Ribosomal protein S23 (rib. prot. S23)	[Genbank:AK012574]	1.3	1	1	4.06E-02	NS	NS
Ribosomal protein L21 (RpL21)	[Genbank:U93863]	0.8	1	1	3.72E-02	NS	NS
t-complex polypeptide 1 (tcp-1)	[Genbank:D10606]	1	1.9	1	NS	5.27E-04	NS
Mannosidase 2, alpha 1 (Man2a1)	[Genbank:XM_123163]	1	1.4	1.5	NS	2.43E-04	5.85E-03
**Mitochondrial**							
Type 6 control region flanking tRNA (mtDNACtrlRNA)	[Genbank:U47435]	0.6	1	1	1.70E-02	NS	NS
tRNA-Thr gene (tRNA-Thr)	[Genbank:AJ313380]	0.6	1.7	1	3.59E-02	2.72E-04	NS
16S ribosomal RNA gene (t16srRNA)	[Genbank:AY011146]	1	0.7	0.8	NS	7.65E-04	2.88E-02
Mouse mitochondrion, complete genome (Mitochond.)	[Genbank:J01420]	1	1.3	1.1	NS	1.42E-03	3.22E-02
Solute carrier family 25 member 3 (Slc25a3)	[Genbank:BC018161]	0.7	1	1	2.38E-02	NS	NS
Cytochrome c oxidase subunit VIIb (Cox7b)	[Genbank:NM_025379]	0.8	1	1	1.60E-02	NS	NS
**Protection against radicals**							
Ferritin heavy chain 1 (Fth1)	[Genbank:NM_010239]	1	1.5	1.3	NS	1.66E-05	4.17E-02
**Organelle transport**							
Homolog rat dynein LIC-2 53/55 (HmlgratDncli2)	[Genbank:NM_031026]	1	1.4	1	NS	9.46E-05	NS
**Reduction of aromatic aldehydes**							
Aldehyde dehydrogenase 3B1 (ALDH3B1)	[Genbank:XM_129078]	1	1.7	1.4	NS	4.17E-04	3.71E-02
**Unknown**							
Clone RP23-465D7 on chromosome 11 (RP23-465D7)	[Genbank:AL591486]	0.6	1	0.7	3.11E-02	NS	1.93E-02
Hypothetical-Binding-protein-dependent transport (Hp)	[Genbank:AK010392]	1.5	1.5	1.5	1.53E-02	3.29E-02	5.59E-03
Clone ct7-25g12 (ct7-25g12)	[Genbank:AC093447]	0.7	1	1	8.92E-04	NS	NS

T1: NOD.B10 treated with BSA, C1: NOD.B10 saline controls, T2: NON treated with BSA, C2: NON saline controls, T3: combined analysis of NOD.B10 and NON treated with BSA, C3: combined analysis of NOD.B10 and NON saline control. NS: Non Significant. Genes found to discriminate between any two of the groups of mice are represented. For each gene, the mean expression value ratio between treated and control animals are represented. Student test p values obtained for significant comparison are also shown.

**Table 2:** Common genes altered by protein overload and diabetes

Gene Name ID	Expression ratios					P values				
	
	T1/C1	T2/C2	T3/C3	D2/C4	D1/C4	T1/C1	T2/C2	T3/C3	D2/C4	D1/C4
tcp-1 [Genbank:D10606]	1	1.9	1	0.7	0.7	NS	5.3E-04	NS	0.002	3.5E-05
FKBP52 [Genbank:X17069]	1	0.8	0.8	0.7	0.8	NS	0.007	0.003	4.5E-05	0.01
VAPA [Genbank:BC003866]	0.7	1	1	0.7	0.7	0.002	NS	NS	7.2E-05	2.5E-04
Cox7b [Genbank:NM_025379]	0.8	1	1	0.7	0.8	0.01	NS	NS	2.4E-05	0.001
mtDNACtrlRNA [Genbank:U47435]	0.6	1	1	1	0.7	0.01	NS	NS	NS	5.9E-05
Slc25a3 [Genbank:BC018161]	0.7	1	1	0.8	1	0.02	NS	NS	0.004	NS
AcylCoAtrsp [Genbank:BC005616]	0.7	1	1	0.6	0.6	0.03	NS	NS	0.005	1.5E-04
RpL21 [Genbank:U93863]	0.8	1	1	0.7	0.8	0.03	NS	NS	5.2E-05	0.001
HistAcetyl [Genbank:AF319620]	0.7	1	0.7	0.7	0.6	0.04	NS	0.02	0.002	2.2E-04
Hmlg.apopts [Genbank:AK008911]	0.9	1	1	0.7	0.8	0.04	NS	NS	5.5E-04	9.6E-05
rib. prot. S23 [Genbank:AK012574]	1.3	1	1	0.5	0.6	0.04	NS	NS	2.8E-05	1.9E-04

T1, T2, T3, treated animals and C1, C2, C3 controls are as in Table (1), NOD long-term diabetic (D2), NOD new-onset diabetic (D1), non-diabetic NOD control (C4). For each gene, the mean expression value ratio between treated (or diabetic) and control animals are represented. Student test p values obtained for every comparison are also shown.
